# Making Lemonade Together -- How Program Directors, Applicants, and Medical Schools Can Thrive During the Upcoming Interview Season

**DOI:** 10.15694/mep.2020.000198.1

**Published:** 2020-09-15

**Authors:** Robert Daulton, Farzaan Kassam, Kevin Milligan, Anna Berry

**Affiliations:** 1 University of Cincinnati College of Medicine; 2 Baylor College of Medicine

**Keywords:** Virtual Interview, Residency Application, Residency Match, Program Director, Medical Student, Medical Schools

## Abstract

This article was migrated. The article was marked as recommended.

The virtual residency interview process ushers in a new era of medical education. Many stakeholders are increasingly concerned as validated recommendations regarding Match success appear less reliable, fossilized rules have become increasingly fluid, and traditional streams of communication have become inadequate. Program directors will look to sell their program using unvalidated methods. Applicants will make life-altering decisions using fewer data points than historically available. Medical schools will endeavor to advise their students as they gear up for breaking new ground. In this piece, we introduce considerations and recommendations for the main players involved in the virtual interview process. If each party prioritizes teamwork and communication, we can collectively tackle the challenges of the upcoming cycle and turn lemons into lemonade.

## Introduction

The upcoming virtual interview season presents new considerations for timeless challenges ( Lee *et al.*, 2019; Hammoud, Standiford and Carmody, 2020). Determining the best institution for one’s graduate medical education is difficult and finding the candidates that best fit a residency program can be just as challenging. This application cycle also creates new expectations for medical schools, who previously played a supporting role in this process once the Medical Student Performance Evaluations were submitted ( Chretien *et al.*, 2015). How do applicants determine “fit” if they have never met their potential future colleagues? How do programs best showcase their culture and city? How do medical schools best prepare their students for success given the logistical and financial disruptions to normal operations created by COVID-19? These are amongst the innumerable questions and considerations that stakeholders face as we embark upon the virtual interview season ( Bird *et al.*, 2019).

Equity must be the bedrock upon which a successful virtual interviewing season is built. Now more than ever, the medical community is acutely aware of the ways in which the Black community, indigenous communities, and other people of color have faced systematic and institutionalized racism in all strata of academia, including medicine. We also recognize that mere recognition is not enough; we must intentionally resist the inequalities that plague our society and too often permeate our medical systems. Our subconscious view of an individual, or their environment, impacts applicants and interviewers more often than we realize ( Maxfield *et al.*, 2019). Those from lower socioeconomic backgrounds may be unable to afford technology used to enhance their virtual representation, and those in rural locations may face unique challenges accessing reliable high-speed internet. Some students fear that implicit biases will be harder to overcome in a virtual interview format. We are passionately in favor of addressing the uncertainties on the horizon with solutions that are steeped in equity.

Despite these challenges, with a bit of good will, and a lot of flexibility, all parties can work together to achieve everyone’s primary goal: a successful Match 2021. Presented below are recommendations from a collaboration of medical students at institutions across the country to help achieve this common aim.

## Considerations for Program Directors

PDs face unique challenges as they aim to overhaul the entire interview season without a playbook. Converting tried and true methods to a new medium will generate unforeseen barriers. PDs will confront additional pressures to enact holistic review methods as they combat racial injustices and the impending elimination of a three digit Step 1 score ( Aibana *et al.*, 2019; Katsufrakis and Chaudhry, 2019; Crane, Chang and Azamfirei, 2020). In line with the need for holistic review, the added salience of racial unrest demands a critical analysis of interview programming. Programs must now double their efforts to identify hidden components of their program or curriculum which may incubate bias or racism ( Lee *et al.*, 2019). As the highest party in the food chain, residency programs will inevitably set the pace for the upcoming interview cycle. Applicants will look to them for continued leadership and reassurance.

### Recommendation 1: Find creative solutions for organic interactions.

Applicants desire opportunities that foster organic interactions with other interviewees, residents, and faculty without the added pressure of being constantly observed ( Kenigsberg *et al.*, 2020). Observing resident camaraderie has historically been a helpful indicator of program culture which is now threatened by the virtual interview ( Kenigsberg *et al.*, 2020; Huntington, Haines and Patt, 2014). This could be resolved by offering multiple avenues for resident engagement highlighting co-resident interactions and allowing applicants to interact with multiple residents. Applicants will look for creative virtual interactions with residents to guide them as they appraise program culture and fit while simultaneously combating virtual fatigue. Potential opportunities to foster these connections include virtual game nights, longitudinal connections by pairing residents with a small cohort of applicants, interactions via social media, optional second look experiences after programs have submitted their rank lists (if safe), and opportunities for current applicants to meet program alumni in their geographic location.

### Recommendation 2: Communicate early and often regarding technology requirements for upcoming virtual interviews.

Prior to the virtual handshake, applicants will need advanced notice of your program’s preferred platform (Zoom, WebEx, Microsoft Teams, etc.) to familiarize themselves with important aspects of the technology such as screen sharing, the chat box, and using the mute button. Consider having program staff host a pre-interview tech check with interviewees a few days before the scheduled interview, similar to the pre-presentation PowerPoint upload which has become commonplace at academic conferences. Similarly, a statement to students about what to do in the event of a connection failurs will go a long way in mitigating the stress of a dropped call. It will be impossible to prevent every mishap, but we can set everyone up for success by anticipating the major tech issues.

### Recommendation 3: Distribute a detailed schedule prior to interview day so applicants can adequately prepare for the time considerations and unique features of your program’s interview experience.

As we enter uncharted waters, more communication from programs will inevitably be required. Will the interview day follow typical business hours? How will programs account for applicants in different time zones? Would it be possible to remain involved with patient care on the day of an interview? Programs should consider hosting different interview days beginning at different times to accommodate applicants in all time zones. Furthermore, the programming start time and length of your interview day should be clearly articulated so that students can adequately prepare. Clear communication will mitigate pre-interview anxiety and allow applicants to focus on what matters most - expressing themselves, sharing their stories, and finding the best fit.

### Recommendation 4: Consider augmenting your digital footprint to connect with applicants through enhanced website design and social media.

Those who are able to leverage the power of technology will surely reap the benefits. A recent study looking at Nephrology fellowship match rates indicated that programs active on social media were more likely to fill their spots ( Matchett, Astor and Maursetter, 2020). Through avenues such as Twitter, applicants will be able to discern much of what they were looking for on interview day - program values, resident life, and the countless intangibles that make up program culture. Inviting residents and program leadership to engage on social media through personal accounts or program account takeovers is an opportunity to display the inner workings of your program and highlight the unique individuals on your team. Furthermore, interactive and well-designed websites can achieve similar goals by helping put a face to the name. Of importance, applicants from minority ethnic and racial backgrounds will look to residency program photos to assess program diversity. Applicants are eager to know that students like them can thrive in your program.

## Considerations for Medical Students

We all miss living in precedented times. Fourth year students are simultaneously adapting to an ever-changing medical education landscape and preparing for an application cycle unlike any other. The anxieties students normally face during this time have been compounded by cancelled away rotations, altered clinical rotations, and the transition to a virtual interview model ( Rose, 2020). Due to significant differences between previous and current application cycles, near-peer advice will not be sufficient to fully mitigate the novel challenges that current applicants face. Students applying across many specialties have unanswered questions. How many letters of recommendations do I need, and how do I obtain them when my specialty clerkship was cancelled? While there is comfort in knowing that these concerns are shared by all applicants, that alone cannot assuage every fear. It is appropriate to take a moment to identify our anxieties and acknowledge that they are a normal reaction to the unknown. Despite the turbulence, we will reach our destination. In lieu of expending precious energy on anxieties that we cannot control, let us take a collective deep breath and shift our focus to doing the things we can. There are steps that we, as students, should all be taking to best set ourselves up for success.

### Recommendation 1: Leverage your wide array of both medical and non-medical networks.

Now is the time to take advantage of the “smallness” of our world by tapping into our far-reaching personal and professional networks. Utilize your school’s alumni network to connect with early and mid-career physicians in your specialty. Throughout your clinical years, you worked with numerous physicians. Consider reaching out to those with whom you really connected, whether they work in your intended field or not. Ask if they have colleagues and friends in your desired specialty with whom you could connect. This is also a great time to join professional medical societies, many of which offer free membership to medical students. This may serve as yet another opportunity to build relationships with fellow applicants, residents, and attendings. Program directors care about connecting with students that genuinely want to be a part of their team. Sharing a genuine interest with your dream program may help jumpstart relationships and, maybe, create a lasting impression. Don’t be afraid to reach out to that MS4 you met once during your first year who is now a resident at your top choice. If you are considering a cross-country move, now is the time to call up that friend from high school who moved to the Bay Area to work for Google. During this time of extended physical distancing, people are hungry for connection, and many will love the opportunity to tell you about their homes and experiences.

### Recommendation 2: Utilize social media to connect with prospective programs and fellow applicants.

One potential benefit of a virtual interview season is that programs are rapidly increasing their social media presence. This class has the opportunity to know more about programs than ever before. This is happening across many platforms, but most notably on Twitter. Take advantage of these opportunities to get an inside look at programs by creating professional accounts on these platforms. To the applicants that fear they don’t have a strong network of personal or professional advocates, Twitter is an outlet for connecting with the medical community ( Chretien *et al.*, 2015). It is important to note that the official code of conduct for social media interaction is far from defined. As such, you should keep in mind that all of your social media engagement is public. Assume that anything you post will be seen by programs that you are interested in.

### Recommendation 3: Communicate early and often with all interested parties regarding your individual circumstances and challenges.

Expecting medical schools and residency programs to know what we need without telling them directly may be unrealistic. As virtual interviews approach, it will be important for medical students to communicate with both their medical school and prospective residency programs about their specific circumstances. Each individual will have unique needs that will only be understood if articulated. If you anticipate any interview day issues, early and respectful communication with program coordinators may help resolve concerns in a timely fashion.

## Considerations for Medical Schools

Historically, students have utilized a combination of peers and institutional advisors to guide them through the residency application process. This year, near-peer advice will be crucially important but also have a limited scope. As such, students will be looking to their medical schools now more than ever. Students recognize that COVID-19 has taken a serious toll on health systems and medical schools around the nation; despite this, medical schools remain uniquely positioned to support students as this application season unfolds. The needs of each individual student and collective student body will vary. These recommendations are not a substitute for soliciting and actively listening to the needs of your students. Presented here are a few broad recommendations to help medical schools as they begin to think about this important work.

### Recommendation 1: Adopt a more hands-on advising approach this cycle, especially for students who do not have a “home program” for their specialty at your institution.

Since the announcement that the interview season would be virtual, PDs around the nation have been working diligently to develop guidance for applicants. Many specialties have released consensus statements communicating these expectations, some have hosted webinars and workshops, and others are still developing guidelines. At schools with home programs, students and advisors alike can connect directly with PDs for specialty-specific guidance. However, for schools that do not have home PDs in a specialty, advisors this year must be intentional and proactive about connecting with program director associations to ensure they have the most up to date guidance for each specialty. Advisors should also create robust outlets for providing frequent updates to students, especially regarding topics such as sub-I requirements, letters of recommendation, and application/interview caps. Establishing a listserv for applicants in each specialty could facilitate targeted and frequent communication between advisors and advisees. One way to capture the unique needs of your student body is to complete a needs assessment by way of a class survey or focus group. Another potential avenue for collecting real time concerns would be to establish a line of communication, be it online or through a student representative, from the student body to faculty. This would allow students to communicate effectively and ensure they have the support they need.

### Recommendation 2: Offer formal training in virtual interview etiquette and provide opportunities for mock interviews.

By providing students with comprehensive interview etiquette training, medical schools can ensure students are getting their information from a trusted source. To avoid reinventing the wheel, schools may be able to look to senior residents for guidance when creating the “Zoom Interview Curriculum.” These individuals may have recently completed virtual fellowship interviews and therefore may have identified answers to common questions. Mock interviews will serve as an additional opportunity for applicants to test their virtual interview space. These “practice runs” will allow interviewees to test Wi-Fi connection, lighting, and video chat etiquette. Offering mock interview opportunities with known and trusted mentors/advisors will ensure that students receive the honest feedback they need to adequately prepare for this new format.

### Recommendation 3: Provide interview accommodations to those who need it.

In an ideal world, each applicant would conduct virtual interviews from the comfort of their home. Unfortunately, not all applicants have a home environment suitable for virtual interviewing. Reasons for this include but are not limited to living in a rural area without high speed internet access, with a partner who works from home, with small children or pets, in a crowded apartment complex, or simply not owning the required technology. To address these disparities, medical schools should consider offering interview space to those students who require an alternative interview location. At many institutions, this may be as easy as providing reservable sound-proofed interview cubicles in a campus building. Not all schools possess such space or will be able to accommodate for an influx of room reservation requests in a socially distanced world. For these schools, consider investigating rental spaces in the local community, such as public libraries, shared working spaces, or even hotels. This may be a costly and logistically challenging feat, so early action and planning may help to avoid last minute stressors. At a minimum, providing applicants with necessary technology and lighting for their homes, as well as standardized virtual interview backgrounds may help mitigate many student concerns.

## Conclusion

After a Spring afflicted by uncertainty, applicants are prepared for much of the same as we approach residency interviews. Despite being a technologically literate applicant pool, anxieties concerning internet failures, and implicit bias have established themselves as prominent concerns shared by applicants. Although it is not a panacea, clear communication may be the antidote for many of the concerns noted above. Applicants will look to programs and home institutions alike for leadership as the book on virtual interviews remains to be written. Program leadership, applicants, and medical schools all want the same thing: a successful Match 2021. By working together, communicating effectively, and emphasizing equity, we can take the lemons at hand and achieve a desirable outcome.

## Take Home Messages

• The upcoming virtual interview season presents new considerations for timeless challenges.
• Program directors will attempt to convert reliable methods to a new format by way of creating unique outlets for virtual connection.
• Although medical students have anxieties about virtual interviews, through networking, meaningful engagement on social media, and expressing concers early, they can thrive in this new format.
• Medical schools will be required to take a more active role in applicant advising as their advisees face innumerable challenges posed by the virtual intervew.
• By working together, communicating effectively, and emphasizing equity, all stakeholders can collectively work towards a successful Match in 2021.

## Notes On Contributors

Robert Daulton: Robert is a fourth year medical student who will be applying to Categorical Pediatrics. He recently finished a research fellowship at Nationwide Children’s Hospital and is also a producer and co-host of the UnsCripted Medicine Podcast. ORCID: https://orcid.org/0000-0001-9469-5306

Farzaan Kassam: Farzaan is a fourth year medical student who will be applying to Urology. He is co-president of the University of Cincinnati College of Medicine Class of 2021. ORCID: https://orcid.org/0000-0002-2093-261X

Kevin Milligan: Kevin is a fourth year medical student who will be applying to Internal Medicine. He is a producer and co-host of the UnsCripted Medicine Podcast and has interests in medical education and oncology.

Anna Berry: Anna is a fourth year medical student who will be applying to Medicine-Pediatrics. She is also enrolled at Duke Divinity School where she is pursuing a Masters in Theological Studies. ORCID: https://orcid.org/0000-0001-6024-4566
