# Supplementary material for: Making Lemonade Together -- How Program Directors, Applicants, and Medical Schools Can Thrive During the Upcoming Interview Season
Source: MedEdPublish (2016). 2020 Sep 15;9:198. [Version 1] doi: 10.15694/mep.2020.000198.1 (PMC10699402; doi:10.15694/mep.2020.000198.1)
Supplement: Peer review history

# Reviewer report: Reviewer response for version 1

**DOI:** 10.21956/mep.20158.r31144

**Published:** 2020-12-10

**Authors:** Ken Masters

**Affiliations:** 1 Sultan Qaboos University

This review has been migrated. The reviewer awarded 3 stars out of 5 An interesting practical tips paper on how to deal with upcoming interviews for residency applicants to medical schools, where applicable. There are, however, some issues that need to be addressed. In most cases, the problems stem from assumptions of contextual knowledge that are not appropriate. The journal is international, and international readers need to be apprised of local conditions, procedures and terminology, otherwise, they have to guess at what is intended. In particular:• The word “residency” should be added to the title, otherwise, the title is too vague.• The process of residency interviews is not ubiquitous, and, where it does occur, does not occur at the same time in every country, so the authors need to give some background as to the country (or countries?) to which this applies, and a little about the process as it stood before COVID-19. This is especially as the authors appear to take for granted that the reader understands the context and the terminology (e.g. what is “the interview season”? What does “Match 2021” mean? Is this a generic term that the authors have created, or is this a known term in the authors’ country? What is the “Bay Area”? and several other issues).• Similarly, the reference to Spring is lost if one does not know the hemisphere of the country. Either stipulate it, or refer to the months.• Also, “PDs” needs to be properly written out and then abbreviated. One can figure it out from the context, but the reader should not be expected to figure things out. It is the authors’ responsibility to conform to the standard.• “We all miss living in precedented times” This is a bold assumption, because, no, actually, some of us don’t :-)• And the “Making Lemonade Together” is a little clichéd, so could probably be dropped So I think this is a useful paper, but many readers are excluded from understanding it because they do not have the contextual knowledge required. I look forward to Version 2 in which these issues are addressed.

Reviewer Expertise:

NA

No decision status is available

# Reviewer report: Reviewer response for version 1

**DOI:** 10.21956/mep.20158.r31145

**Published:** 2020-09-20

**Authors:** Subha Ramani

**Affiliations:** 1 Harvard Medical School, Brigham and Women's Hospital

This review has been migrated. The reviewer awarded 4 stars out of 5 The Covid-19 pandemic has created problems at all levels of education and a variety of health professions. While many educators have discussed in depth the challenges and solutions related to virtual teaching and learning, the authors of this paper discuss a challenging interview season coming up. The process of resident recruitment and selection of newly graduated medical students into various residency training programs need a radically different approach during an unprecedented time. The result of residency selection changes the professional trajectory of many a young doctor. Not only does it throw challenges in the path of residency program directors and selection committees, but equally challenging is the process of selection of programs by new junior doctors. Ensuring equity and diversity would be an important goal of the interview season.The authors propose useful recommendations to ensure that program directors and interviewers communicate the spirit of their program, the institutional culture and the work climate adequately in a virtual format. Important recommendations pertain to detailed communication about the platform, connectivity requirements, opportunities for applicants to engage with residents, scheduling the interview day across different time zones, mechanisms to deal with technological failures etc. A virtual interview season also requires better websites and engagement with social media to engage young applicants. Student applicants should be reassured that selection will be fair and equity will be addressed in a virtual environment and residency program leaders must train all their faculty and resident interviewers in allaying anxieties of applicants and presenting a friendly and open demeanour despite the restrictions of the electronic platforms.All health professions educators who are in a position to interview applicants for any position will find these recommendations useful.

Reviewer Expertise:

NA

No decision status is available
